# Influence of Perioperative Variables on Surgical Site Infection in Patients With Frailty and Prefrailty Undergoing Coronary Artery Bypass Grafting: Retrospective Multicenter Study

**DOI:** 10.2196/79408

**Published:** 2026-10-16

**Authors:** Mauricio Guerrieri, Omar Mejia, Bianca Meneghini, Natali Rodrigues, William Burato, Maisa Barros, Daniel Wernsbach, Lucas Bartelega, Alexandre Hueb, Fabio Jatene

**Affiliations:** 1 Medical School, Universidade de São Paulo, Av. Dr. Enéas Carvalho de Aguiar, 44, Cerqueira César, São Paulo, 05403-900, Brazil, 55 35 988168825; 2 Medical School, Universidade do Vale do Sapucaí, Pouso Alegre, Brazil

**Keywords:** coronary disease, coronary artery bypass, surgical wound infection, aged, frailty

## Abstract

**Background:**

Surgical site infection (SSI) in patients with frailty undergoing myocardial revascularization is a major clinical challenge, and there is a gap in the literature related to this topic.

**Objective:**

This study aimed to identify perioperative variables associated with SSI in patients with frailty and prefrailty undergoing coronary artery bypass grafting within 30 days of the procedure.

**Methods:**

This retrospective observational study included 467 patients with frailty or prefrailty aged 60 years and older who underwent coronary artery bypass grafting in 6 Brazilian centers between January 2018 and December 2024. Frailty was defined using the Fried criteria. SSI was defined as a wound with exposed tissue with positive culture requiring antibiotic treatment. Univariate and multivariate analyses were carried out.

**Results:**

The rate of SSI was 5.6% (26/467). The New York Heart Association classification (*P*=.02), Charlson Comorbidity Index (CCI; *P*=.01), use of cardiopulmonary bypass (CPB; *P*=.003), CPB time (*P*=.02), total surgery time (*P*<.001), occurrence of bleeding (*P*=.04), mechanical ventilation time (*P*=.001), length of hospital stay (*P*<.001), intensive care unit stay (*P*<.001), and need for reoperation (*P*<.001) variables were associated with SSI in unadjusted comparisons. In the multivariate model, the CCI (odds ratio 1.58, 95% CI 1.25‐1.99; *P*<.001) and use of CPB (odds ratio 4.89, 95% CI 1.71‐13.94; *P*=.003) were independently associated with SSI.

**Conclusions:**

The CCI and the use of CPB were independently associated with SSI.

## Introduction

Longevity is increasing in many countries, where life expectancy at birth is rising [1]. A reduction in mortality among older age groups seems to be one of the main reasons for this increase [2], and consequently, living with chronic, incurable diseases is becoming more frequent [3]. Population aging, improved public health policies, and advances in cardiovascular disease prevention and treatment have increased the number of people living with chronic disease because more individuals survive acute cardiovascular events, increasing the prevalence and absolute number of older adults living with these conditions and their complications [4]. This compromises the quality of life of these individuals and highlights the concept of frailty, which is gaining prominence in clinical practice [5]. Frailty is the physiological decline of the body’s functional reserves [5,6].

Aging-associated frailty increases the risk of postoperative complications, and age and frailty are recognized as risk factors for mortality and morbidity in patients undergoing surgical procedures [5,7,8]. Coronary artery bypass grafting (CABG) is a well-established, common procedure; specifically, 169,705 operations were performed in 2020 in the United States and 19,125 procedures in Brazil’s Unified Health System in 2023 [9,10]. Given these figures, it is essential to consider the morbidity associated with this procedure and seek better clinical outcomes and quality of life for patients.

Among the postoperative complications that arise within 30 days, surgical site infection (SSI) stands out for its clinical relevance, occurring in 0.3% to 3.5% of myocardial revascularization cases [11-16]. The associated mortality rate varies from 10% to 47% in cases of SSI, significantly contributing to an increase in morbidity, mortality, and hospital costs and potentially trebling the procedure’s cost and having a considerable impact on public resources [15,17-19].

SSI is associated with worse short- and long-term clinical outcomes. Interestingly, age alone does not seem to be a relevant predictive factor for this outcome [20-22]. Moreover, frailty has not been properly investigated as a possible risk factor for SSI in patients undergoing myocardial revascularization.

It is well established that frailty increases morbidity and mortality in older adults as well as the risk of postoperative complications by up to 2.54 times [5,7]. Therefore, it is plausible that individuals with frailty may be more susceptible to SSI than the general population. There may be additional factors associated with this potential increase in risk, which, if identified, will facilitate the development of specific preventive strategies for this patient population.

The objective of this study was to identify the perioperative variables associated with SSI in patients with frailty and prefrailty undergoing CABG within 30 days after surgery.

## Methods

### Study Design and Participants

This was a retrospective, longitudinal secondary analysis of data from the multicenter randomized FRAGILE (Off-Pump Versus On-Pump Coronary Artery Bypass Grafting in Frail Patients) trial, in which enrolled participants were randomly assigned to undergo either on-pump (cardiopulmonary bypass; CPB) or off-pump CABG surgery in 6 Brazilian hospitals [23]. Data were collected using the FRAGILE study dataset with standardized variable definitions across centers; however, some inter-institutional variation in clinical practice and documentation cannot be excluded. In the FRAGILE study, CPB use was assigned through randomization rather than surgeon preference; therefore, CPB was not subject to confounding by indication at the point of treatment allocation.

Although the FRAGILE protocol initially planned for the participation of 10 centers, the final dataset comprised 6 centers due to incomplete data capture and reduced recruitment at some sites during the COVID-19 pandemic period. The present secondary analysis used the final FRAGILE dataset to preserve data completeness and integrity. The data were extracted in March 2025 after the research ethics committee approved the secondary analysis and waived the requirement to obtain new informed consent from the participants.

This study included data from 467 patients aged 60 years and older with 2 or more Fried frailty criteria who underwent on- or off-pump CABG between January 2018 and December 2024 [5]. As a retrospective secondary analysis of an existing multicenter study, the sample size was determined by data availability over the study period rather than an a priori calculation. Patients who underwent urgent and emergency surgery and combined cardiac surgery, those with creatinine clearance below 30 mL per minute and hemoglobin A_1c_ above 8.5%, and those with a history of cardiac surgery were excluded because these conditions are strong determinants of infectious complications and perioperative morbidity and could introduce substantial confounding. Missing data were minimal for the variables used in the final model; therefore, a complete-case analysis was performed. Frailty was assessed based on the Fried criteria, which include unintended weight loss, self-reported exhaustion, weakness, slowness, and low physical activity. Patients with 2 criteria were considered prefrail, whereas those with 3 or more criteria were deemed to be frail. We included both patients with prefrailty and frailty because the FRAGILE study defined vulnerability using 2 or more Fried criteria. We acknowledge that inclusion of participants with prefrailty may reduce discrimination compared with a frail-only cohort and interpreted the frailty score accordingly.

SSI was operationally defined using predefined criteria captured in the FRAGILE dataset aligned with Society of Thoracic Surgeons–related documentation of clinically significant sternal wound complications:

- An open wound with exposure of muscle or subcutaneous tissue or need for reoperation due to infection [24]
- Positive culture unless the patient was taking antibiotics at the time of sample collection for bacterial culture [24]
- Treatment with antibiotics in addition to perioperative prophylaxis [24]

The primary outcome was the occurrence of any SSI within 30 days after CABG. All recorded events—including sternal dehiscence, superficial SSI, deep SSI, and mediastinitis—were combined into a single composite SSI outcome. Each event was also classified by subtype for descriptive reporting only. Subtype-specific analyses were not performed because of the limited number of events in each category.

The preoperative variables analyzed were age, sex, BMI, diabetes mellitus, creatinine clearance, New York Heart Association (NYHA) functional class, Charlson Comorbidity Index (CCI), and frailty score. Intraoperative variables included the number of mammary arteries used, CPB use, and CPB and surgery duration. The secondary outcomes included postoperative variables such as occurrence of bleeding, duration of mechanical ventilation, duration of hospitalization, and duration of intensive care unit (ICU) stay. SSI was assessed within 30 days after surgery. Length of hospital stay refers to the total hospitalization time of patients who developed SSI, including the period after SSI diagnosis and subsequent treatment during the same hospitalization. Potential collinearity between CPB time and surgery duration was considered during model building; to preserve model parsimony and avoid redundancy, only the clinically most informative variables were retained in the final adjusted model.

The CCI is a weighted index comprising 19 comorbid conditions and was originally developed to estimate mortality risk [25]. Higher scores indicate a greater comorbidity burden. In this study, the CCI was analyzed as a continuous variable.

Postoperative variables were included for exploratory association analysis and should not be interpreted as preoperative risk predictors. This analysis was performed with the aim of understanding the distribution, trends, and patterns of each variable in isolation and in relation to the occurrence of SSI. Although additional intraoperative complexity variables were available in the FRAGILE dataset, including left ventricular ejection fraction, number of distal anastomoses, bilateral mammary artery use, CPB time, and cross-clamp time, these variables were not all included in the final multivariable model because of the limited number of SSI events. To reduce the risk of overfitting and unstable estimates, the final model was kept parsimonious and focused on variables with stronger statistical and clinical relevance.

### Statistical Analysis

Statistical analyses compared baseline and perioperative characteristics between patients with and without SSI within 30 days after CABG. Secondary postoperative outcomes (bleeding, mechanical ventilation duration, ICU stay, and hospital length of stay) were also compared between groups. The continuous variables were initially assessed for normal distribution using the Shapiro-Wilk test and expressed as medians and IQRs owing to the asymmetrical distribution of the data. The Mann-Whitney *U* test for nonparametric data was used to compare 2 independent groups: occurrence of infection and no infection.

The categorical variables were expressed as absolute and relative values and compared between groups using the Pearson chi-square test. The Fisher exact test was used in the presence of expected frequencies of less than 5 in one or more cells of the contingency table. In the case of categorical variables with multiple ordinal categories, such as the number of mammary arteries used, the likelihood ratio test was used. Univariable screening may fail to retain important confounders that are not individually significant, which may bias adjusted estimates in sparse-event settings. Additionally, multiple logistic regression analysis was performed to identify the factors independently associated with the occurrence of postoperative outcomes. The variables with statistical significance in the univariate analysis (*P*<.05) and those considered clinically relevant were included in the model. The results were expressed as odds ratios (ORs) with their corresponding 95% CIs and *P* values. Analyses were performed according to randomized treatment allocation using CPB assignment as the exposure. The level of statistical significance set for all analyses was 5% (*P*<.05), and the analyses were carried out using the SPSS software (version 19; IBM Corp).

### Ethical Considerations

The study protocol was approved by the Research Ethics Committee of Hospital das Clínicas, Faculdade de Medicina, Universidade de São Paulo, Brazil (Certificate of Presentation for Ethical Consideration 30702020.0.0000.0068; approval number 4.228.709; approved on August 21, 2020). This study was conducted in accordance with Brazilian National Health Council Resolution 466/2012 and the ethical principles of the Declaration of Helsinki.

Because this was a retrospective study based on the analysis of previously collected clinical data and medical records, the requirement for individual informed consent was waived by the Research Ethics Committee. All data used in the study were coded and deidentified before analysis. Access to the research database was restricted to the authorized research team, and no information capable of identifying individual participants is presented in the manuscript. Participants received no financial or other compensation for inclusion in this study.

## Results

The sample consisted of 467 patients, 26 (5.6%) of whom had SSI. The classification of the type of SSI was as follows: 1.9% (n=9) sternal dehiscence, 1.5% (n=7) superficial SSI, 1.1% (n=5) deep SSI, 0.4% (n=2) deep infection with use of corticosteroids, 0.4% (n=2) mediastinitis, and 0.2% (n=1) subtype not specified in the database (Table 1). The classification of “deep infection with corticosteroid use” reflects the original FRAGILE dataset subtype classification; subtype categories were reported descriptively only and were not used in the univariable or multivariable models.

Table 2 shows the results related to the preoperative variables associated with SSI.

The median age was similar between the groups with and without infection (70, IQR 65‐74 vs 69, IQR 65‐74 years, respectively; *P*=.79), as was the median BMI (28, IQR 24‐33 vs 26, IQR 24‐29 kg/m^2^, respectively; *P*=.07). Between the 2 groups, there were no statistically significant differences in sex (male: 14/26, 53.8% vs 303/441, 68.7%, respectively; *P*=.12), presence of diabetes mellitus (10/26, 38.5% vs 153/441, 34.7%, respectively; *P*=.70), or creatinine clearance (median 63, IQR 48‐82 vs 62, IQR 51‐76 mL/min, respectively; *P*=.91).

**Table 1.** Classification of types of surgical site infection (N=467).

Type of surgical site infection	Events, n (%)
Sternal dehiscence	9 (1.9)
Superficial surgical site infection	7 (1.5)
Deep surgical site infection	5 (1.1)
Deep surgical site infection with corticosteroid use	2 (0.4)
Mediastinitis	2 (0.4)
Subtype not specified	1 (0.2)

**Table 2.** Preoperative variables in patients with and without surgical site infection (SSI).

Variables	Infection		*P* value
	No (n=441)	Yes (n=26)	
Age (y), median (IQR)	69 (65-74)	70 (65-74)	.79^a^
Sex (male), n (%)	303 (68.7)	14 (53.8)	.12^b^
BMI (kg/m^2^), median (IQR)	26 (24-29)	28 (24-33)	.07^a^
Diabetes mellitus, n (%)	153 (34.7)	10 (38.5)	.70^b^
Creatinine clearance (mL/min), median (IQR)	62 (51-76)	63 (48-82)	.91^a^
NYHA^c^ class, n (%)			.02^d^
I	184 (41.7)	8 (30.8)	
II	149 (33.8)	6 (23.1)	
III	77 (17.5)	5 (19.2)	
IV	31 (7.0)	7 (26.9)	
Charlson Comorbidity Index, median (IQR)	4 (3-5)	5 (4-6)	.01^a^
Frailty score, median (IQR)	4 (3-4)	4 (3-5)	.49^a^

a Mann-Whitney *U* test.

b Pearson chi-square test.

c NYHA: New York Heart Association.

d Likelihood ratio test.

However, there was a significant association between NYHA functional class and the occurrence of infection (*P*=.02), with a higher proportion of patients in class IV in the group with infection (7/26, 26.9% vs 31/441, 7.0%; Figure 1). The CCI was higher among patients with infection (median 5, IQR 4‐6 vs 4, IQR 3‐5; *P*=.01; Figure 2). The frailty score was slightly higher in the infection group than in the group without infection but without statistical significance (median 4, IQR 3‐5 vs 4, IQR 3‐4, respectively; *P*=.49). Table 3 shows the results obtained for the intraoperative variables.

The number of mammary arteries used also showed no statistically significant difference between the infection and no infection groups (*P*=.52; ie, using only 1 mammary artery [20/26, 76.9% vs 298/441, 67.6%, respectively] or using 2 mammary arteries [6/26, 23.1% vs 133/441, 30.2%, respectively]). The use of CPB was more frequent in the group with SSI (21/26, 80.8% vs 223/441, 50.6%; *P*=.003; Figure 3). CPB time was statistically associated with SSI in univariable analysis (median 85, IQR 71‐128 vs 85, IQR 65‐105 minutes in the infection and no infection groups, respectively; *P*=.02). Although the medians were identical, the distribution differed between groups (IQR shift), and this finding should be interpreted cautiously. Additionally, the total time of the surgical procedure was different between the groups, with prolonged surgery time being associated with a higher occurrence of infection (median 303, IQR 250‐359 vs 270, IQR 217‐325 minutes in the infection and no infection groups, respectively; *P*<.001). Table 4 shows the results obtained for the postoperative variables.

Occurrence of bleeding was higher in the group with infection (2/26, 7.7% vs 4/441, 0.9%; *P*=.04). The duration of mechanical ventilation was significantly longer among patients with infection (median 18, IQR 8‐21 vs 8, IQR 5‐14 hours; *P*=.001), as were the total length of hospital stay (median 41, IQR 23‐55 vs 7, IQR 6‐11 days; *P*<.001) and the length of ICU stay (median 5, IQR 4‐15 vs 3, IQR 2‐5 days; *P*<.001). The need for reoperation was considerably more frequent in the group with infection (13/26, 50% vs 13/441, 2.9%; *P*<.001).

In the multiple logistic regression analysis, 2 variables were identified as independently associated with SSI (Table 5). All 467 patients were included in the multivariable regression.

**Figure 1.**
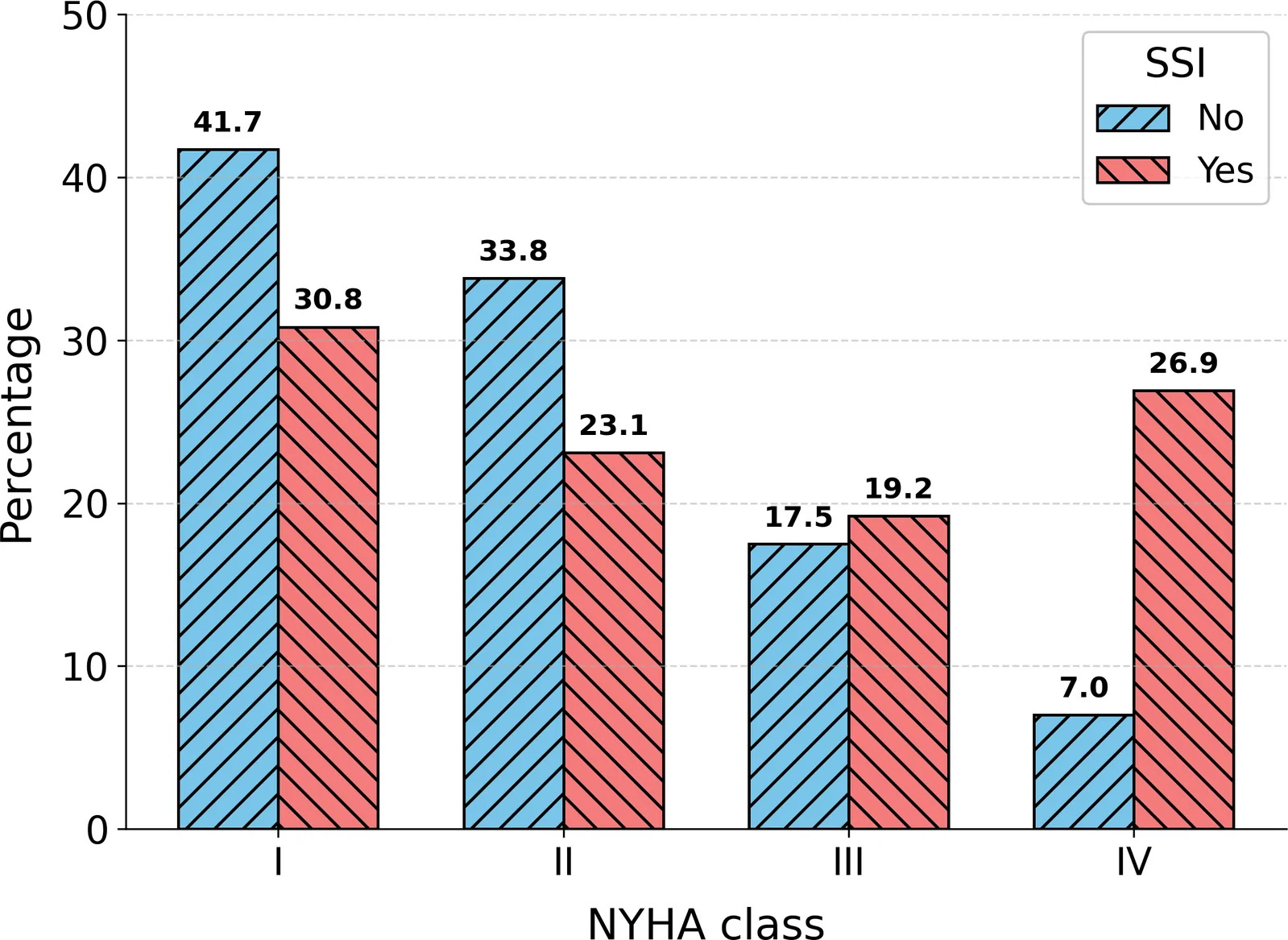
Distribution of postoperative infection according to New York Heart Association (NYHA) classification.

**Figure 2.**
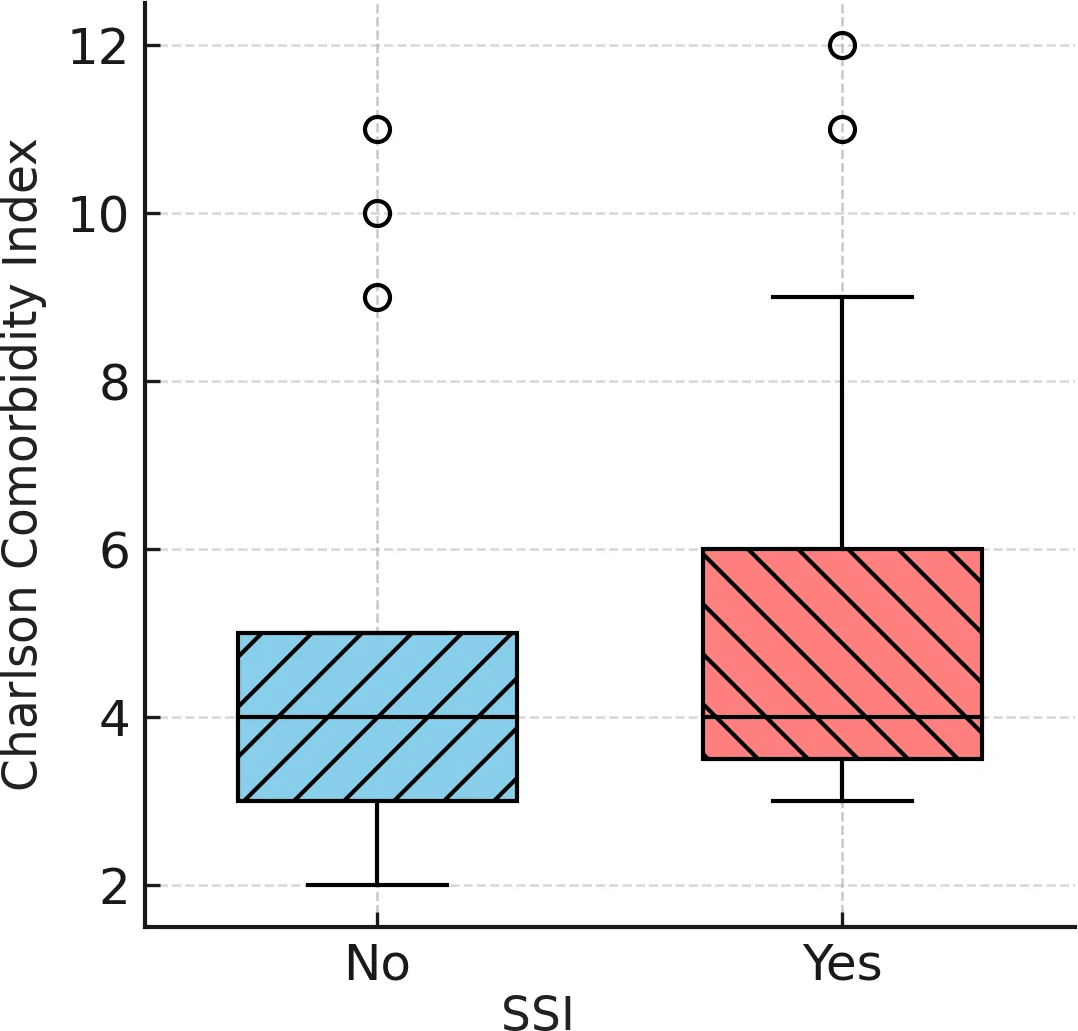
Comparison of the Charlson Comorbidity Index between patients with and without postoperative infection.

**Table 3.** Intraoperative variables in patients with and without postoperative infection.

Variables	Infection		*P* value
	No (n=441)	Yes (n=26)	
Number of mammary arteries, n (%)			.52^a^
0	10 (2.3)	0 (0)	
1	298 (67.6)	20 (76.9)	
2	133 (30.2)	6 (23.1)	
Cardiopulmonary bypass, n (%)	223 (50.6)	21 (80.8)	.003^a^
Cardiopulmonary bypass time (min)^b^, median (IQR)	85 (65-105)	85 (71-128)	.02^c^
Total surgery time (min), median (IQR)	270 (217-325)	303 (250-359)	<.001^c^

a Pearson chi-square test.

b Cardiopulmonary bypass duration was evaluated only among patients who underwent cardiopulmonary bypass (223 patients without surgical site infection and 21 patients with surgical site infection).

c Mann-Whitney *U* test.

**Figure 3.**
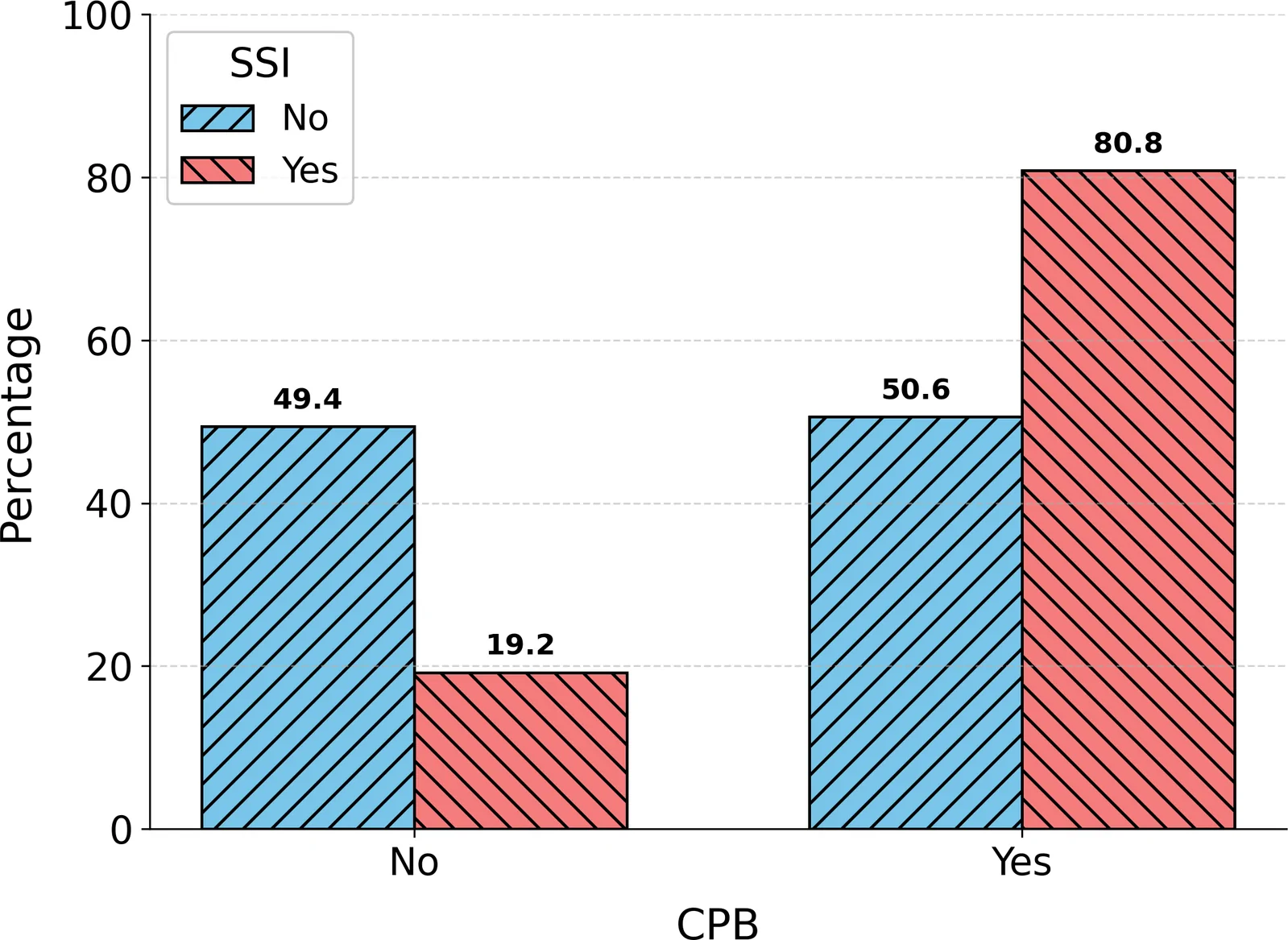
Distribution of postoperative infection according to the use of cardiopulmonary bypass (CPB) during the procedure.

**Table 4.** Results for the postoperative variables in patients with and without postoperative infection.

Variables	Infection		*P* value
	No (n=441)	Yes (n=26)	
Bleeding, n (%)	4 (0.9)	2 (7.7)	.04^a^
Mechanical ventilation duration (h), median (IQR)	8 (5-14)	18 (8-21)	.001^b^
Length of stay (d), median (IQR)	7 (6-11)	41 (23-55)	<.001^b^
Length of stay in the ICU^c^ (d), median (IQR)	3 (2-5)	5 (4-15)	<.001^b^
Reoperation during hospitalization, n (%)	13 (2.9)	13 (50.0)	<.001^a^

a Fisher exact test.

b Mann-Whitney *U* test.

c ICU: intensive care unit.

**Table 5.** Multiple logistic regression for factors independently associated with postoperative surgical site infection.

Variables	β coefficient^a^ (SE)^b^	OR^c^ (95% CI)	*P* value
Charlson Comorbidity Index	0.46 (0.12)	1.58 (1.25-1.99)	<.001
Cardiopulmonary bypass	1.59 (0.54)	4.89 (1.71-13.94)	.003

a Log-odds scale.

b Constant=–5.96 (SE 0.84).

c OR: odds ratio.

The CCI was associated with the outcome (OR 1.58, 95% CI 1.25‐1.99; *P*<.001), indicating a 58% increase in the likelihood of infection with each additional point on the index. The use of CPB also proved to have a significant association (OR 4.89, 95% CI 1.71‐13.94; *P*=.003). The model showed good discrimination, with an area under the receiver operating characteristic curve of 0.752 (95% CI 0.662‐0.842; *P*<.001; Figure 4).

**Figure 4.**
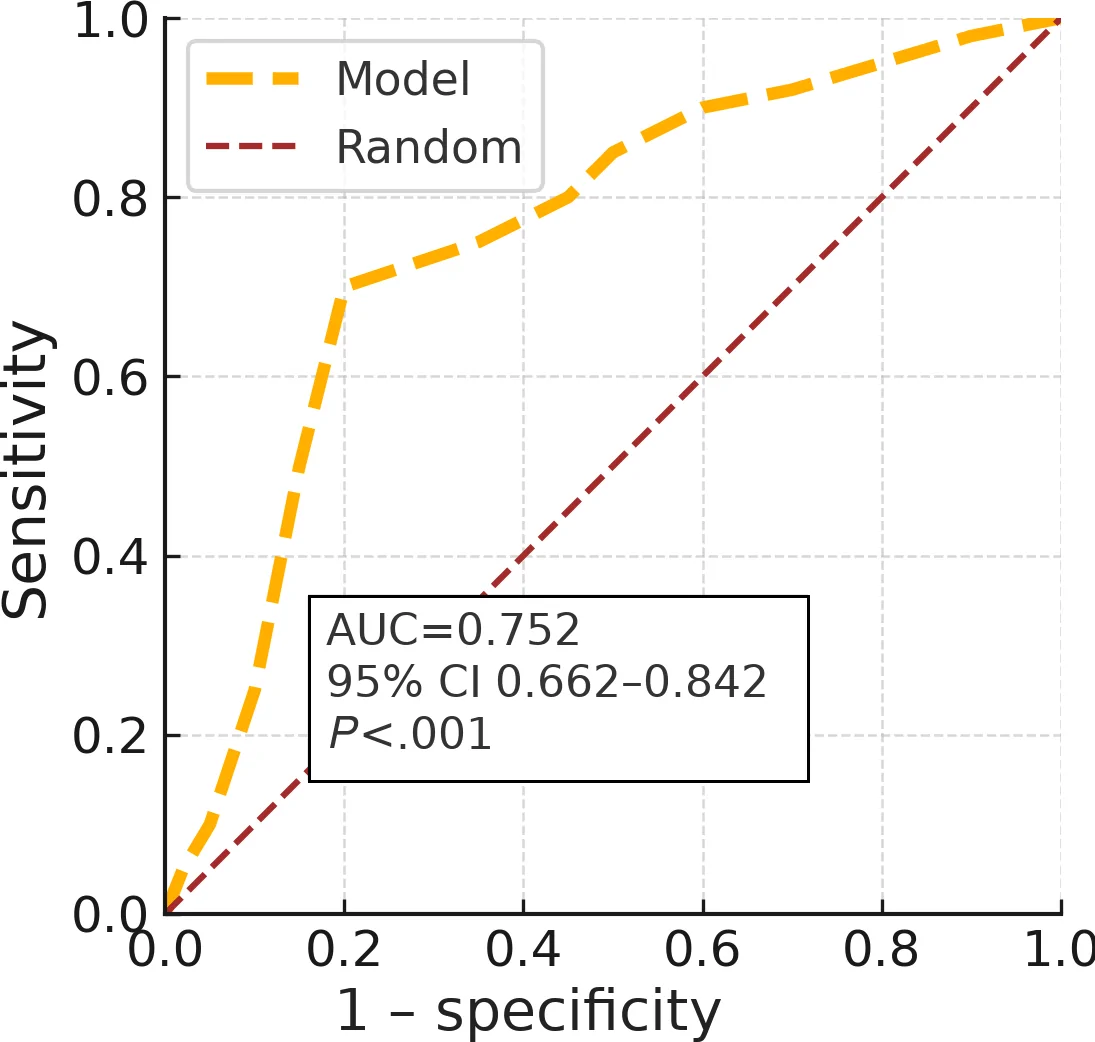
Receiver operating characteristic (ROC) curve of the predictive model for postoperative infection in high-risk surgical patients. AUC: area under the ROC curve.

## Discussion

In this study, an SSI rate of 5.6% (26/467) was observed in patients with frailty undergoing myocardial revascularization. Although this was higher than the rates reported in previous studies [16,20-22], no study has addressed frailty in a population like the one we studied. Among the factors analyzed, the CCI and the use of CPB were identified as associated with SSI, especially CPB, which was independently associated with higher odds of SSI.

The association between frailty and adverse outcomes in surgery has been widely described. Studies have shown that patients with frailty have a higher risk of mortality, prolonged hospitalization, and infection complications [5,7,26]. In this study, even after adjusting for other variables, the CCI was significantly associated with the occurrence of SSI (OR 1.58, 95% CI 1.25‐1.99; *P*<.001), which demonstrated that the accumulation of comorbidities associated with a decline in physiological reserves increases the association with SSI by 58% for each 1-point increase in the CCI. We additionally clarified how frailty was operationalized in the cohort and how its distribution may have limited discrimination within an already frail population.

The frailty score did not discriminate SSI risk within this cohort, likely because participants were selected for frailty and prefrailty by design, reducing variability in frailty burden and limiting discrimination for this end point. Rather than functioning as a within-cohort predictor, frailty in this study should be viewed as a biological substrate of vulnerability potentially linked to reduced immune responsiveness and diminished physiological reserve that may contribute to the susceptibility to SSI. Consequently, additional perioperative factors may better explain variation in SSI occurrence among patients already meeting frailty and prefrailty criteria.

The use of CPB also showed a significant association with infection, corroborating the literature that associates CPB with greater systemic inflammatory activation, increased tissue permeability, and transient immune suppression [27,28]. The same trend was observed for CPB time, which differed between the groups. The use of this technique and the intensity of the associated stress are important markers of operative complexity and greater exposure to infectious risks. Mechanistically, CPB is associated with complement activation and cytokine release, endothelial and glycocalyx injury, and transient immune dysfunction, which may impair wound healing and facilitate infection in vulnerable patients.

Reoperation was strongly associated with SSI in the unadjusted analysis. However, because reoperation was recorded after CABG and its temporal relationship with SSI diagnosis was not reported, it should be interpreted as a postoperative correlate rather than as a predictor of SSI. This finding corroborates those of studies showing that surgical revision, especially at an early stage, compromises anatomical barriers and favors colonization of deep tissue of the thorax [29]. Additionally, patients who require reoperation often have more severe clinical profiles and are subject to repeated stressors, including the use of invasive devices for prolonged periods.

Although variables such as age, BMI, and diabetes mellitus have traditionally been related to sternal infection [16], they did not prove to be significant in this study. This result appears to be associated with the fact that the analysis was specific to a population with frailty, in which factors associated with a drop in physiological reserve are more prominent.

Other relevant variables were NYHA functional class and length of mechanical ventilation, which were significantly associated with infection, similar to what has been described in the literature [17,30,31]. More advanced functional classes, such as NYHA class IV, showed a higher association with infection, possibly because they reflect greater previous hemodynamic compromise. This leads to longer intubation time in the postoperative period, which can hinder healing and predispose patients to infection.

The number of mammary artery grafts used did not influence the occurrence of infection; however, there are studies that demonstrate an increase in incidence when 2 mammary grafts are used [32,33]. The results of our study may encourage surgeons, when choosing grafts, to use a greater number of mammary grafts because of the benefit of durability and because the complications of SSI are similar whether one or more is used.

Length of hospital and ICU stay were associated with a higher occurrence of SSI, which indicates a negative cycle in which longer hospital stay increases the association with infection and the occurrence of infection increases the length of hospital stay. Longer hospital stays are associated with longer use of invasive devices, more contact with hospital pathogens, and changes in the patient’s microbiota. In turn, the occurrence of infection contributes to an increase in hospital admissions, the use of antibiotics, the need for procedures secondary to infection, and delay in postoperative recovery. Consequently, guidelines associated with multiple actions have emerged with the aim to reduce the length of hospital stay and, subsequently, reduce the occurrence of SSI [34].

Makary et al [7] suggested incorporating frailty score into the set of risk predictors commonly used in clinical practice. Risk assessment in cardiovascular surgery is carried out using scores such as the EuroSCORE and the Society of Thoracic Surgeons score, which estimate risk without considering the decline in the individual’s physiological reserves. The inclusion of the frailty score and prior assessment of comorbidities during the evaluation contribute to the early identification of patients who are more susceptible to complications, allowing for preventive actions through adjustments to the surgical technique, preoperative preparation of the patient’s physical condition, and strict control of comorbidities.

This multicenter study used standardized criteria for assessing frailty and multivariable logistic regression, which showed that the CCI and CPB use were independently associated with SSI. Preoperative recognition of patients with frailty with a high number of comorbidities, especially those undergoing CPB, can guide prevention strategies and more intensive monitoring in the pre- and postoperative periods.

However, this study had some limitations. The number of SSI events was relatively small, which may have limited the stability of the multivariable model and increased the risk of overfitting. The retrospective use of medical records from multiple centers may have resulted in variation in the completeness and consistency of documentation for certain clinical variables, particularly SSI subtype, microbiological findings, and antibiotic treatment. Because of the limited number of SSI events, we did not adjust for center-level effects, and residual confounding due to interinstitutional variation in perioperative care and SSI surveillance cannot be excluded. The composite SSI end point included events with different clinical severity (superficial infection, deep infection, mediastinitis, and dehiscence). Because of the limited number of events in each subtype, we were unable to perform robust subtype-specific modeling; thus, associations may differ across SSI phenotypes. We assessed discrimination (area under the receiver operating characteristic curve) but did not formally evaluate model calibration; therefore, the clinical applicability of predicted risks should be interpreted cautiously. Although CPB assignment was randomized in the parent trial, the present analysis included only centers with complete eligible data; therefore, postrandomization exclusions and center selection may have introduced selection bias and limited generalizability. We did not formally quantify multicollinearity using the variance inflation factor or correlation coefficients; therefore, residual collinearity among operative variables cannot be excluded.

We conclude that factors such as NYHA functional class IV, CCI, CPB time, bleeding, duration of mechanical ventilation, need for reoperation, use of CPB, and length of hospital and ICU stay showed an unadjusted association with SSI. Additionally, we demonstrated that the CCI and the use of CPB were independently associated with SSI in the multivariable model within 30 days after CABG.

## Abbreviations

CABG: coronary artery bypass grafting
CCI: Charlson Comorbidity Index
CPB: cardiopulmonary bypass
FRAGILE: off-pump versus on-pump coronary artery bypass grafting in frail patients
ICU: intensive care unit
NYHA: New York Heart Association
OR: odds ratio
SSI: surgical site infection
